# 1540. Becoming SexuWELL: Optimization of a Sexual Health Reminder to Promote Pre-Exposure Prophylaxis (PrEP) Education and Consultation within the Orlando Veterans Affair Healthcare System (OVAHCS)

**DOI:** 10.1093/ofid/ofad500.1375

**Published:** 2023-11-27

**Authors:** Minh Q Ho, Cierra N Brewer, Nicholas Scapelito, Katrina Mclean, Linda Chia, Matthew Cole, William Gage, Karen Slazinski

**Affiliations:** Orlando VA Healthcare System, 14014 Deep Forest Court, Florida; VHA, Chicago, Illinois; Orlando VAHCS, Kissimmee, Florida; Orlando VAHCS, Kissimmee, Florida; VA, Bellevue, Washington; VA Capital Health Care Network (VISN 5), Veterans Health Administration, Huntsville, Alabama; Washington DC VA medical Center, Washington, District of Columbia; Orlando VA Healthcare System, 14014 Deep Forest Court, Florida

## Abstract

**Background:**

Sexual wellness is an important the Veteran Affairs (VA) whole health approach. Obtaining accurate and timely sexual health histories serve to identify risks of acquiring viral and bacterial communicable disease.

Specifically, U.S. Department of Health and Human Services (HHS) launched the Ending the HIV Epidemic in the U.S. (EHE) initiative in 2019 with the aim to reduce new HIV infections in the U.S. by 90% by 2030 through scaling up key HIV prevention and treatment strategies. One key prevention is increasing utilization of Pre-Exposure Prophylaxis (PrEP). A multidisciplinary team implemented sexual wellness screen using clinical reminders at Orlando VA Health Care System (OVAHCS).

**Methods:**

A clinical reminder was created in the electronic health record (eHR) to alert primary care teams if a patient had not been screened for sexual health practices in the last year. Nurses are prompted to ask patients if they are open to discussing sexual health history to reduce their risk of infection. Health factor data (HFD) is transmitted from the eHR to VA’s Corporate Data Warehouse (CDW) on a nightly basis. Structured query language is used to extract HFD from the CDW to aggregate patient responses to the sexual health practice questions. Then a retrospective chart review was conducted on patients that completed a sexual health reminder and stated “yes” to learning more about a medication that can prevent HIV.

Sexual Health History (SHH) Clinical Reminder Questions

**Figure:**
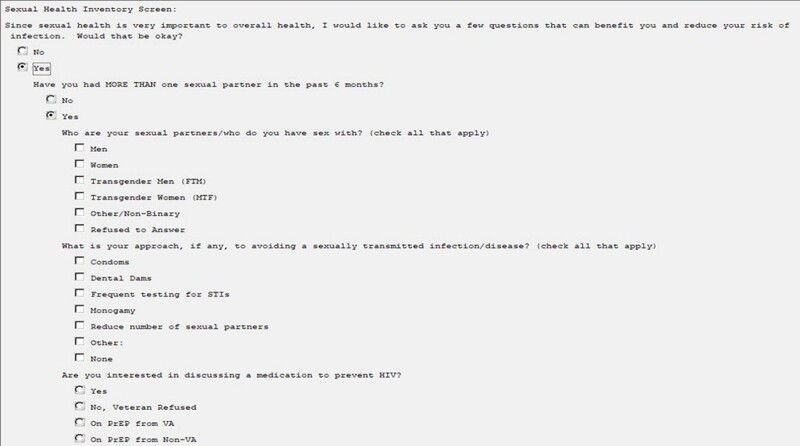

These are some of the questions asked by nursing team when a Veteran is being screened on the day of the visit, based off of CDC 5Ps.

Microsoft Power BI Report

**Figure:**
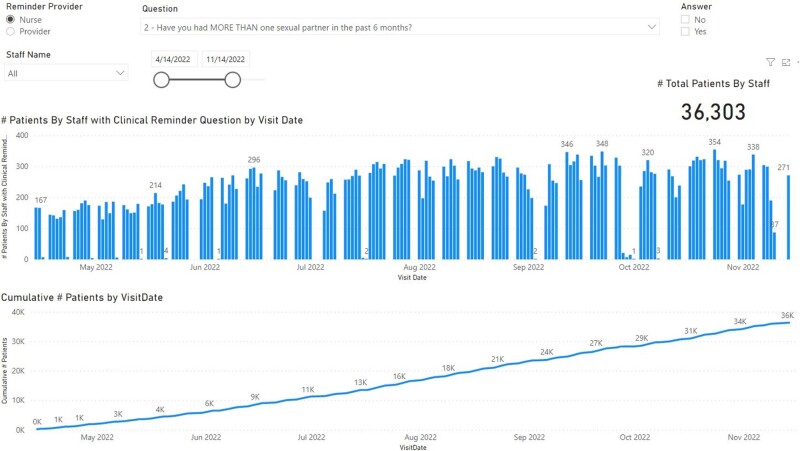

This report displays information from 04/14/22 to 11/14/22. Information such as how many Veterans have been screened , rates of acceptance for discussing sexual health history, and rate of acceptance for an infectious disease consult.

**Results:**

36,303 patients completed the reminder between 04/14/22 and 11/14/22. 189 agreed to referral to discuss PrEP, and 27 were initiated on therapy (14.3%). 18 of the 27 received a second PrEP Rx for a 67% continuation rate. Of those who agreed to discuss, 13% were females, 35% were older than 45 years, and 39% were black. The reminder resulted in an increase in referrals to infectious disease by 143% from 2022 and 278% from 2021. It also increased PrEP prescription by 61% from 2022 and 248%. Retention rate on PrEP with reminder is 60%.

**Figure:**
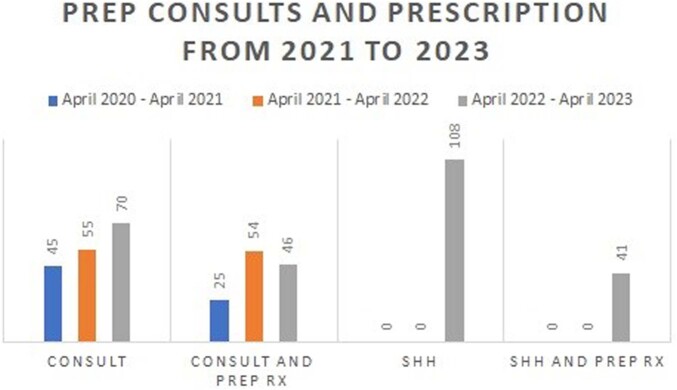

This graph shows the number of PrEP consults and corresponding PrEP prescription from 2020 to 2022. Sexual Health History (SHH) clinical reminder was implemented in 2022 and had an additive effect on the overal increase of PrEP consults and prescriptions.

Race Characterization of Veterans Answering Clinical Reminder

**Figure:**
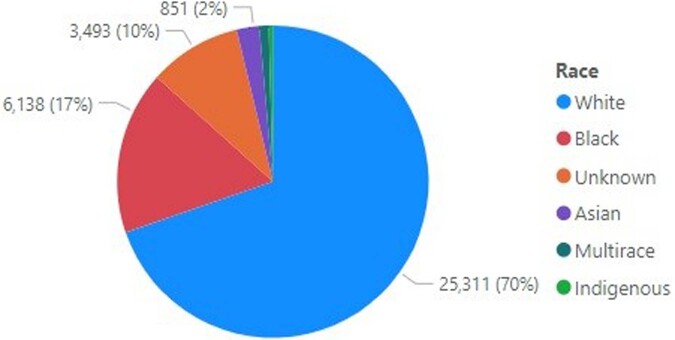

This chart provides a breakdown by Race of who said "Yes" to having more than one sexual partners

Age Characterization of Veterans Answering Clinical Reminder

**Figure:**
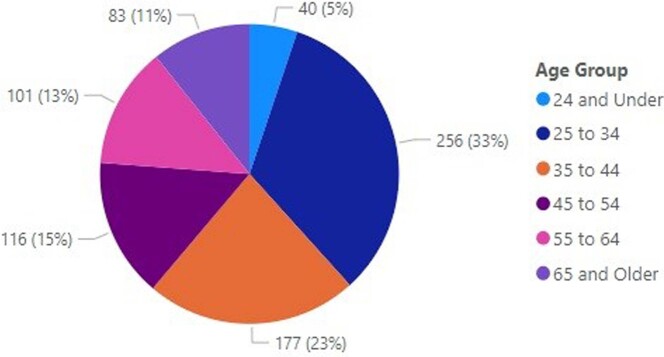

This chart provides a breakdown by Age of who said "Yes" to having more than one sexual partners

**Conclusion:**

The implementation of a sexual health reminder can increase awareness about PrEP and resulted in an increase in consults for PrEP Areas of improvement include ensuring consults are completed and scheduling of patients within 60 days of the consult being accepted. Overall, utilization of clinical reminders work to improve sexual health education and PrEP access.

Pathway to PrEP Initiation

**Figure:**
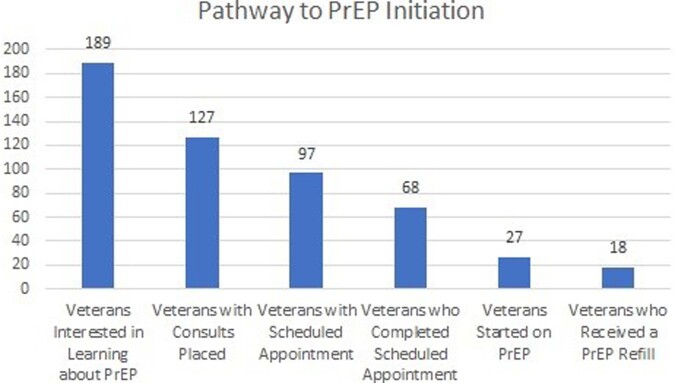

This graph illustrates the pathway from initial number of Veteran who agreed to discuss about PrEP to number who actually got a PrEP prescription and the subsequent second prescription.

Number of Veterans Receiving a PrEP Refill

**Figure:**
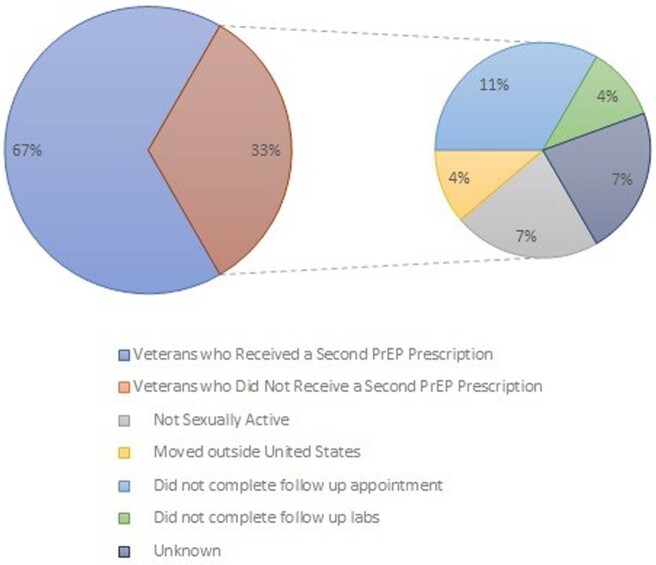

This pie chart describes the number of Veterans who received a PrEP refill as well as reason for no refill

**Disclosures:**

**All Authors**: No reported disclosures

